# Acute Flaccid Myelitis

**DOI:** 10.21980/J8MP9G

**Published:** 2022-07-15

**Authors:** Dane Zappa, Linda L Herman

**Affiliations:** * Vituity Healthcare and Medical Staffing Services, St Agnes Medical Center, Department of Emergency Medicine, Fresno, CA; ^ Sutter Roseville Medical Center, Department of Emergency Medicine, Roseville, CA

## Abstract

**Audience:**

Emergency medicine residents and medical students on emergency medicine rotation.

**Introduction:**

Although a somewhat rare disease, acute flaccid myelitis (AFM) can cause death, and for those pediatric patients that survive, less than 10% have full recovery.^1^ A cluster of cases that resembled polio was first described in California in 2012.^2^ After 120 cases of the disease were confirmed in 2014 in a 5-month period, the Centers for Disease Control and Prevention (CDC) began surveillance of the disease. Since surveillance began, clusters of cases have occurred in a biennial pattern, usually late summer and early fall. There were 218 cases between 2015 to 2017, 238 cases in 2018, 47 cases in 2019, and 32 in 2020.^3^,^4^,^5^ AFM has become recognized as a global disease with cases reported across many countries.^1^ The CDC has noted that the most common location of the first medical encounter of pediatric patients presenting with AFM is the emergency department in every year that surveillance occurred.^5^ Most of the children that are diagnosed with AFM are admitted to the hospital and of those admitted, 30% require intubation.^5^ Deaths related to AFM are due to respiratory involvement and complications. With appropriate recognition and supportive care, mortality can be avoided.

**Educational Objectives:**

At the end of this oral board session, examinees will: 1) demonstrate the ability to obtain a complete pediatric medical history, 2) demonstrate an appropriate exam on a pediatric patient including a neurological exam, 3) investigate the broad differential diagnoses for neuromuscular weakness in a pediatric patient, 4) order the appropriate evaluation studies including an MRI, 5) interpret the use of a negative inspiratory force in determining the need for intubation and level of care upon admission, and 6) demonstrate effective communication with parents and caregivers.

**Educational Methods:**

Oral board case following a standard American Board of Emergency Medicine-style case in a tertiary care hospital with access to all specialists and resources needed. This case was tested using 5 resident volunteers ranging from PGY 1 – 3 in an ACGME (Accreditation Council for Graduate Medical Education)-accredited emergency medicine program. Also, approximately 3 – 5 observers (other residents and medical students) were present during the presentation. Learners were immediately able to provide feedback during the debriefing of the case.

**Research Methods:**

Immediate Feedback was solicited from the learners and observers participating in the case both by verbal discussion and completion of a rating for the case following the debriefing. The efficacy of the educational content was assessed by comparing scoring measures across residents based on the training year. Scoring measures of the ACGME core competencies were performed using a scale from 1–8, 1–4 being unacceptable performance and 5 – 8 being acceptable. Efficacy was assumed based on full completion of the case by the residents who acted as practice oral board candidates, and a debriefing session followed to discuss the key components of the case.

**Results:**

Practice candidates were 1 PGY1 level, 2 PGY2 Level and 2 PGY3 level residents. All residents that were practice candidates anchored on the diagnosis of Guillain-Barré Syndrome (GBS) but despite the anchoring were able to manage the patient appropriately and safely. The average score for practice candidates per level was: PGY1: 5.1, PGY2: 5.8, and PGY3: 6.5. The critical action missed by the PGY1 resident was ordering a negative inspiratory force and one PGY2 did not completely order all of the spinal MRI. All learners, both practice candidates and observers rated the case as 4.2 (1 – 5 Likert scale, 5 being excellent).

**Discussion:**

The educational content effectiveness was two-fold. The content was effective for teaching the presentation and appropriate evaluation to diagnose AFM. AFM is significantly like the presentation of poliomyelitis which originally occurred in sporadic cluster outbreaks and then the number of cases in the United States doubled every 4 – 5 years from 1940 – 1952.^6^ AFM is a disease that occurs in a biennial pattern and needs to be recognized and reported appropriately. The case also encouraged the cognizance of other etiologies for acute neuromuscular weakness in a pediatric patient which may require different diagnostic evaluation and medical management. AFM’ster clinical presentation generally involves asymmetric weakness and may occur in either an ascending or descending pattern with the nadir to maximum weakness attained in a few days. Although there are variants of GBS, the neuromuscular weakness is usually symmetrical and occurs in an ascending pattern with the nadir being reached in 1 – 2 weeks.^7^ AFM requires an MRI with specific abnormalities to meet the case definition while an MRI can be performed when GBS is suspected, but it is not necessary. AFM MRI abnormalities demonstrate brainstem and spinal cord lesions with a predominance of gray matter affected while a GBS MRI demonstrates ventral root abnormalities without any spinal cord or brainstem lesions. Without the MRI results, a patient may be assigned the incorrect diagnosis upon admission. A lumbar puncture and electromyography are required for the diagnosis of GBS. The cerebrospinal fluid (CSF) of GBS demonstrates high protein levels and white blood cell count (WBC) < 10 cells/mm^3^ while the CSF of AFM demonstrates pleocytosis although usually < 100 cells/mm^3^.^1^,^8^ Electromyography will be abnormal in both GBS and AFM, but the test is not necessary for the diagnosis of AFM as it is with GBS. Both intravenous immunoglobulin (IVIG) and plasma exchange shorten the recovery time of GBS while there is no recommendation for treatment of AFM at this time. IVIG, plasmapheresis, and steroids have been utilized with unclear benefits.^1^,^8^ Physical therapy and occupational therapy appear to be significantly important in AFM with nerve transfer surgery as a possibility of cure to areas of muscles that have not recovered significant function in AFM.^9^ Reaching the appropriate diagnosis allows the emergency medicine physician to communicate more accurately with worried parents, providing them with correct information on treatment and progression of the disease.

**Topics:**

Pediatric weakness, pediatric neurologic disorders, acute flaccid myelitis, Gullian-Barré Syndrome, neuromuscular weakness.

## USER GUIDE

**Table t1-jetem-7-3-o1:** 

**List of Resources:**	
Abstract	1
User Guide	4
For Examiner Only	6
Oral Boards Assessment	13
Stimulus	16
Debriefing and Evaluation Pearls	28


[Table t1-jetem-7-3-o1]

**Learner Audience:**

Medical students, interns, junior residents, senior residents

**Time Required for Implementation:**

Case: 15 minutes if used as a single case, approximately 10 minutes if part of a triple case

Debriefing: 5 minutes (when using the oral boards for practice), 10 minutes (when using oral boards case for teaching)

**Learners per instructor:**

Recommend one learner per instructor if using as an oral board case, may have other learners present to observe.

**Topics:**

Pediatric weakness, pediatric neurologic disorders, acute flaccid myelitis, Gullian-Barré Syndrome, neuromuscular weakness.

**Objectives:**

By the end of this oral boards case, examinees will be able to:

1. Demonstrate the ability to obtain a complete pediatric medical history.
2. Demonstrate an appropriate exam on a pediatric patient including neurological exam.
3. Investigate the broad differential diagnoses for neuromuscular weakness which include AFM, Guillain-Barré syndrome (GBS), spinal cord stroke, demyelinating myelitis, poliomyelitis, other infectious myelitis, acute plexopathy, periodic paralysis, botulism, toxic synovitis.
4. Order the appropriate evaluation studies including an MRI.
5. Interpret the use of a negative inspiratory force in determining the need for intubation and level of care upon admission.
6. Demonstrate effective communication with parents and caregivers.

### Linked objectives and methods

The presented case asks learners to care for a pediatric patient with an acute neurologic disorder. In order to effectively diagnose and treat the patient, they will need to understand that this patient is sick, solicit a full history from the patient’s parent, and transition care to the inpatient side. The oral boards format worked best for this case because any patient that presents with acute weakness and respiratory compromise most commonly creates an anchor (particularly in learners) to Guillain-Barré Syndrome. Utilizing the oral boards format and the associated time limit allows learners to use this anchor while still treating the patient successfully, and then discuss other similar causes of neuromuscular pathology with respiratory decline that should be considered.

### Recommended pre-reading for instructor

- None, review references as needed

### Results and tips for successful implementation

This case was presented at a residency program that practices oral boards with all residents semi-annually. It is best utilized after learners understand the basic format of oral boards, the time constraints, and what resources are available to them. We did not test this case on interns who had no experience with an oral boards case. This case was tested on five learners with approximately 3 – 5 resident or medical student observers in each group. Results were highly similar, with learners treating the patient as a GBS case and managing appropriately. Modifications were made to the case following its testing to make the case more classic for AFM with differentiation from other etiologies such as GBS. Feedback was mixed: most of the learners enjoyed the case and appreciated the learning points regarding acute flaccid myelitis as another item on their updated differential, while some of the learners felt misled to GBS. Our suggestion is that if the learner diagnoses and treats this patient as a GBS patient, at the end of the case make sure to recognize that they did so correctly, even if the diagnosis was incorrect. Emphasize that the purpose of this case is to expand the differential list, and that it was not designed to trick the learner. This is a unique opportunity to expand a real differential diagnosis list while avoiding harm in even a theoretical patient.

## Supplementary Information



## Figures and Tables

**Table t2-jetem-7-3-o1:** 

**Vitals:**	HR 108	BP 95/62	RR 22	Temp 37°C	O_2_ Sat 94%

**Table t3-jetem-7-3-o1:** 

**Weight:** 20.4 kg (45 lbs)	**Height:** 45 inches

**Table t4-jetem-7-3-o1:** 

	Milestone	Did not achieve level 1	Level 1	Level 2	Level 3
**1**	**Emergency Stabilization (PC1)**	□ **Did not achieve Level 1**	□ **Recognizes abnormal vital signs**	□ **Recognizes an unstable patient, requiring intervention** **Performs primary assessment** **Discerns data to formulate a diagnostic impression/plan**	□ **Manages and prioritizes critical actions in a critically ill patient** **Reassesses after implementing a stabilizing intervention**
**2**	**Performance of focused history and physical (PC2)**	□ **Did not achieve Level 1**	□ **Performs a reliable, comprehensive history and physical exam**	□ **Performs and communicates a focused history and physical exam based on chief complaint and urgent issues**	□ **Prioritizes essential components of history and physical exam given dynamic circumstances**
**3**	**Diagnostic studies (PC3)**	□ **Did not achieve Level 1**	□ **Determines the necessity of diagnostic studies**	□ **Orders appropriate diagnostic studies** **Performs appropriate bedside diagnostic studies/procedures**	□ **Prioritizes essential testing** **Interprets results of diagnostic studies** **Considers risks, benefits, contraindications, and alternatives to a diagnostic study or procedure**
**4**	**Diagnosis (PC4)**	□ **Did not achieve Level 1**	□ **Considers a list of potential diagnoses**	□ **Considers an appropriate list of potential diagnosis** **May or may not make correct diagnosis**	□ **Makes the appropriate diagnosis** **Considers other potential diagnoses, avoiding premature closure**
**5**	**Pharmacotherapy (PC5)**	□ **Did not achieve Level 1**	□ **Asks patient for drug allergies**	□ **Selects an appropriate medication for therapeutic intervention, considering potential adverse effects**	□ **Selects the most appropriate medication(s) and understands mechanism of action, effect, and potential side effects** **Considers and recognizes drug-drug interactions**
**6**	**Observation and reassessment (PC6)**	□ **Did not achieve Level 1**	□ **Reevaluates patient at least one time during the case**	□ **Reevaluates patient after most therapeutic interventions**	□ **Consistently evaluates the effectiveness of therapies at appropriate intervals**
**7**	**Disposition (PC7)**	□ **Did not achieve Level 1**	□ **Appropriately selects whether to admit or discharge the patient**	□ **Appropriately selects whether to admit or discharge** **Involves the expertise of some of the appropriate specialists**	□ **Educates the patient appropriately about their disposition** **Assigns patient to an appropriate level of care (ICU/Tele/Floor)** **Involves expertise of all appropriate specialists**
**22**	**Patient centered communication (ICS1)**	□ **Did not achieve level 1**	□ **Establishes rapport and demonstrates empathy to patient (and family)** **Listens effectively**	□ **Elicits patient’s reason for seeking health care**	□ **Manages patient expectations in a manner that minimizes potential for stress, conflict, and misunderstanding.**
**23**	**Team management (ICS2)**	□ **Did not achieve level 1**	□ **Recognizes other members of the patient care team during case (nurse, techs)**	□ **Communicates pertinent information to other healthcare colleagues**	□ **Communicates a clear, succinct, and appropriate handoff with specialists and other colleagues** **Communicates effectively with ancillary staff**

**Table t5-jetem-7-3-o1:** 

**#1**	**Patient Information Form**
**#2**	**CBC**
**#3**	**BMP**
**#4**	**Urinalysis**
**#5**	**Venous Blood Gas**
**#6**	**Creatine Kinase**
**#7**	**Thyroid Panel**
**#8**	**COVID and Influenza Testing**
**#9**	**Chest Radiograph**
**#10**	**MRI Cervical, Thoracic, and Lumbar Spine with and without contrast**
**#11**	**CSF Studies**

**Table t6-jetem-7-3-o1:** 

**Patient’s Name:**	**Trevor Riggin**	
**Age:**	**6 years**	
**Gender:**	**Male**	
**Chief Complaint:**	**Weakness**	
**Person Providing History:**	**Mother**	
**Vital Signs:**		
**Temp:**	**37°C**	
**BP:**	**95/62**	
**P:**	**108**	
**RR:**	**22**	
**Pulse**	**Ox: 94%**	
**Weight:**	**20.4 kg (45 lbs)**	**Height: 45 inches**

**Table t7-jetem-7-3-o1:** 

**White blood cell count (WBC)**	**8.0 ×1000/mm3**
**Hemoglobin (Hgb)**	**12.5 g/dL**
**Hematocrit (Hct)**	**37.5%**
**Platelets**	**250 ×1000/mm3**
**Segs**	**64%**
**Bands**	**3%**
**Lymphocytes**	**0%**
**Monocytes**	**31%**
**Eosinophils**	**1%**

**Table t8-jetem-7-3-o1:** 

**Sodium**	**140 mEq/L**
**Potassium**	**3.9 mEq/L**
**Chloride**	**101 mEq/L**
**Bicarbonate**	**35 mEq/L**
**Blood Urea Nitrogen (BUN)**	**11 mg/dL**
**Creatinine (Cr)**	**0.9 mg/dL**
**Glucose**	**95 mg/dL**

**Table t9-jetem-7-3-o1:** 

**Color**	**Yellow**
**Appearance**	**Clear**
**Sp Gravity**	**1.010**
**pH**	**6.0**
**Glucose**	**Negative**
**Protein**	**Negative**
**Ketones**	**Negative**
**Leukocyte Esterase**	**Negative**
**Nitrite**	**Negative**
**Blood**	**Negative**
**Microscopy**	
**White blood cells (WBC)**	**0–1/hpf**
**Red blood cells (RBC)**	**0–2/hpf**
**Squamous cells**	**0/hpf**
**Bacteria**	**0/hpf**

**Table t10-jetem-7-3-o1:** 

**pH**	**7.33**
**pCO2**	**52 mmHg**
**pO2**	**30 mmHg**
**O2 sat**	**75%**

**Table t11-jetem-7-3-o1:** 

**CK**	**22 U/L**	**Reference 22 – 198 U/L**

**Table t12-jetem-7-3-o1:** 

**TSH**	**1.52 mU/L**	**Reference 0.4 – 4.5 mU/L**
**Total T3**	**146 ng/dL**	**Reference 100 – 200 ng/dL**
**Free T4**	**.98 ng/dL**	**Reference 0.9 – 1.8 ng/dL**

**Table t13-jetem-7-3-o1:** 

**COVID**	**Not detected**
**Influenza A**	**Not detected**
**Influenza B**	**Not detected**

**Figure f1-jetem-7-3-o1:**
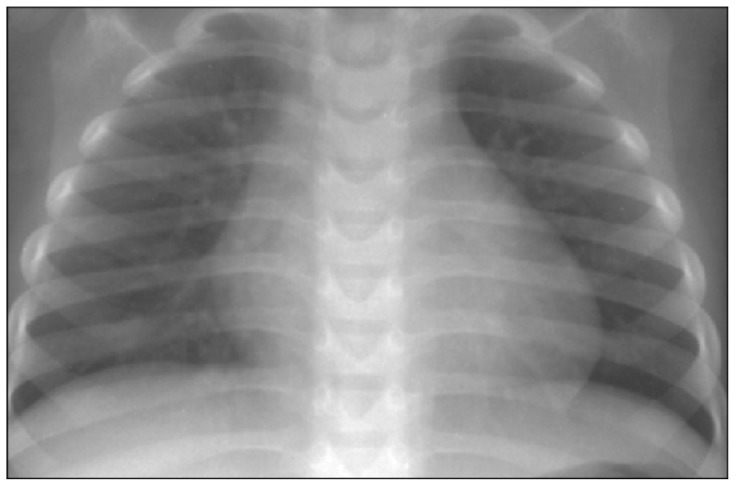
Image Source: Author’s own image.

**Table t14-jetem-7-3-o1:** 

**Opening pressure:**	**10cm H2O**
**Color:**	**Clear**
**Clarity:**	**Clear**
**WBC:**	**56 cells**
**RBC:**	**0 cells**
**Protein:**	**41 mg/dL**
**Glucose:**	**50 mmol/L**
**Gram stain:**	**Negative**
**Other:**	**Negative**

## FOR EXAMINER ONLY

### Oral Case Summary

**Diagnosis: Acute Flaccid Myelitis**

**Case Summary:** A 6-year-old male without known chronic medical illnesses presents to the ED brought in by mother complaining that he has difficulty walking and less use of the right arm. The symptoms began two days ago and have been progressively worsening. He has never experienced this in the past. He had upper respiratory symptoms with rhinorrhea and a nonproductive cough 5 days ago. At that time, he ate normally, did not have a fever, and there were no GI symptoms. The exam reveals an asymmetric upper and lower extremity weakness, diminished deep tendon reflexes with preserved sensation in the lower extremities, preserved deep tendon reflexes and sensation in the upper extremities. The patient requires an evaluation for the neurological findings which includes laboratory, MRI, and lumbar puncture. The most significant complication is respiratory compromise, so the learner needs to order a negative inspiratory force (NIF) and interpret correctly. The patient does develop respiratory compromise during the stay in the ED, and the learner will have to intubate.

**Order of Case:** Patient arrives by private vehicle accompanied by the mother. The patient is triaged, and vital signs are performed. The learner then obtains the history from the mother which should include childhood immunization history and past medical illnesses including recent upper respiratory infection symptoms. If the learner does not ask the mother about prior illnesses, the nurse or ED technician should state, “Did the mother mention that he was sick recently?” The physical exam is then performed and includes assessment of upper and lower extremity strength, deep tendon reflexes, sensation, and ambulation. If the learner does not perform a complete exam, the patient should tell the nurse that he has to go to the restroom, and mother states that he is too weak to walk there. After the history and examination, the learner should order appropriate diagnostic testing and also request that the respiratory therapist perform a NIF. A normal NIF of −32 cm H20 is obtained and the patient is placed on oxygen. The laboratory evaluation should include CBC, BMP, blood culture, thyroid panel, CK, VBG or ABG, CXR, urinalysis, COVID swab, influenza testing. The learner should realize that this is a spinal cord disorder and not order a CT of the brain. If the learner does order a CT, the nurse should ask, “what do you think that the CT will show?” The learner should order an MRI (with and without contrast) of the cervical, thoracic, and lumbar spinal cord. While waiting for the MRI to be performed, the learner should perform a lumbar puncture (LP), explaining risks and benefits to the mother. If the learner does not perform the LP, the nurse should tell the learner, “MRI called and said that they are backed up. Is there anything that you want to do while we are waiting?” Upon returning from MRI, the patient complains of shortness of breath. He now is tachypneic and hypoxic. The learner may ask for a repeat NIF or decide to intubate. The patient is placed on a ventilator. The results of the diagnostic evaluation return and the learner makes the diagnosis, admits to the PICU, and places a neurology consult.

**Disposition:** Admission to Pediatric Intensive Care Unit (PICU)

**Critical Actions:**

1. Performs complete physical examination including strength, deep tendon reflexes, and ambulation.
2. Obtains a Negative Inspiratory Force (NIF)
3. Orders cervical, thoracic, and lumbar spinal cord MRI with and without contrast
4. Performs lumbar puncture after explaining risks and benefits to mother
5. Intubates the patient
6. Admits patient to the PICU

### Historical Information

**Chief Complaint:** Weakness

**History of present illness:** 6-year-old male without known chronic medical illnesses, uncomplicated birth, previously ambulatory, presents with difficulty with ambulation. Had an upper respiratory infection 5 – 7 days prior to arrival. No other positive review of systems. Mother first noted two days ago when he was unsteady but now having difficulty standing when getting up. Mother also noted that he has been favoring his right arm and using only his left to eat and pick up objects. He had complained of pain in the right arm before he began to favor the arm, but he told her that the pain was gone.

**Past Medical history:** No past medical history, immunizations up-to-date

**Past Surgical history:** None

**Patient’s Medications:** Occasional acetaminophen

**Allergies:** None

**Social history:**

- Tobacco: None
- Alcohol: No tobacco use, no exposure to secondhand smoke in the home
- Drug use: None

**Family history:** Mother and father are both healthy, no siblings

### Physical Exam Information

[Table t2-jetem-7-3-o1][Table t3-jetem-7-3-o1]**General appearance:** Disheveled, mild-to-moderate distress

**Primary survey:**

- **Airway:** Intact
- **Breathing:** Breath sounds clear bilaterally
- **Circulation:** No gross signs of hemorrhage or blood loss, peripheral pulses palpable and symmetrical, capillary refill less than 2 seconds

**Physical examination:**

- **General appearance:** Well-developed, well-nourished male, no acute distress although appears uncomfortable
- **Head, eyes, ears, nose and throat (HEENT):**
  ○ **Head:** atraumatic, normocephalic (within normal limits)
  ○ **Eyes:** Pupils equal and reactive (within normal limits)
  ○ **Ears:** TMs are clear (within normal limits)
  ○ **Nose:** Within normal limits
  ○ **Oropharynx/Throat:** Moist mucus membranes, pharynx clear, no cracked lips (within normal limits)
- **Neck:** No lymphadenopathy, no thyromegaly (within normal limits)
- **Chest:** Equal chest rise but shallow, clear breath sounds, diminished in the bases
- **Cardiovascular:** Normal heart sounds, regular, peripheral pulses palpable, and symmetrical, capillary refill less than 2 seconds (within normal limits)
- **Abdominal/GI:** Active bowel sounds, soft, nontender, no hepatomegaly, no masses (within normal limits)
- **Genitourinary:** Within normal limits
- **Rectal:** Within normal limits
- **Extremities:** Scattered small ecchymoses anterior tibial areas (if learner asks, mother states that he received them from falling), no edema, no erythema
- **Back:** Within normal limits
- **Neuro:** Cranial nerves II – XII are intact; diminished strength lower extremities – 3/5, unable to bear weight on lower extremities; unable to ambulate; Right upper extremity (RUE): strength – 4/5; Left upper extremity (LUE): strength – 5/5; Reflexes: RUE and LUE: Brachioradialis and biceps tendon – 2+; Lower extremities – patellar and Achilles tendon – absent; sensation intact to all extremities
- **Skin:** Warm and dry, ecchymoses as described, no rashes
- **Lymph:** Within normal limits
- **Psych:** Alert, oriented, cooperative, appropriate (within normal limits)

### Critical Actions and Cueing Guidelines

1. **Critical Action 1**: *Performs complete physical examination including strength, deep tendon reflexes and ambulation.*
  Patient care
  This critical action is met by the learner performing the exam and obtaining the information of asymmetric weakness, preservation of deep tendon reflexes in the upper extremities but not the lower extremities, sensation preservation in all extremities, and difficulty with standing and ambulating.
  Cueing Guideline (if applicable): If the learner does not collect that history, the nurse should tell the learner that the patient tried to walk to the bathroom, could not bear weight on either leg for more than 20 seconds, and she had to take him in a wheelchair, and the mother placed him on the toilet.
2. **Critical Action 2**: *Obtain a Negative inspiratory force (NIF).*
  Patient care, medical knowledge
  This critical action is met by the learner requesting that the respiratory therapist perform a NIF and interpreting it to be normal. Normal NIF is less than −30 cm H_2_O. The original NIF is −32 cm H_2_0. The learner should recognize that −32 cm H2O is within the normal range for a NIF but needs to be followed closely with serial measurements. The learner may or may not choose to place the patient on oxygen.
  Cueing Guideline (if applicable): If the learner fails to order oxygen or measure an NIF, the mother should mention, “I thought maybe that he was having trouble breathing at home.”
3. **Critical Action 3**: *Orders cervical, thoracic, and lumbar spinal cord MRI with and without contrast.*
  Patient care, medical knowledge
  This critical action is met by the learner recognizing an acute neuromuscular process and requesting an MRI of the cervical and thoracic spinal cord.
  Cueing Guideline (if applicable): If the learner does not request an MRI, then the nurse should ask the learner if any tests should be ordered. If the learner decides to order a CT scan, the nurse should ask “What do you think the CT will show?”
4. **Critical Action 4**: *Performs lumbar puncture after explaining risks and benefits to mother.*
  Patient care, medical knowledge, interpersonal and communication skills
  This critical action is met by the learner consenting the mother for a lumbar puncture, providing adequate analgesia and performing lumbar puncture.
  Cueing Guideline (if applicable): If the learner does not perform the LP, the nurse should tell the learner, “MRI called and said that they are backed up. Is there anything that you want to do while we are waiting?”
5. **Critical Action 5**: *Intubates the patient*
  Patient care, medical knowledge
  This critical action is met by the learner recognizing that the patient’s respiratory status is deteriorating and intubating the patient.
  Cueing Guideline (if applicable): If the learner does not intubate the patient, then the respiratory therapist should ask, “What would you like me to do?” Each strategy that the learner tries such as high-flow oxygen, BiPap, etc. does not result in decreasing the patient’s respiratory distress or improving the pulse oximeter.
6. **Critical Action #6:** *Admits patient to the PICU.*
  Patient care, systems-based practice, interpersonal and communication skills
  This critical action is met by the learner giving report to the pediatric intensivist.
  Cueing Guideline (if applicable): If the learner does not admit the patient, the nurse should remind the learner that all the results are back and that a disposition needs to occur.

## ORAL BOARDS ASSESSMENT

*Acute Flaccid Myelitis*

Learner: _________________________________________

**Critical Actions:**

□ Performs complete physical examination including strength, deep tendon reflexes, and ambulation. (patient care)
□ Obtains a Negative Inspiratory Force (NIF) (patient care, medical knowledge)
□ Orders cervical, thoracic, and lumbar spinal cord MRI with and without contrast (patient care, medical knowledge)
□ Performs lumbar puncture after explaining risks and benefits to mother (patient care, medical knowledge, interpersonal and communication skills)
□ Intubates the patient (patient care, medical knowledge)
□ Admits patient to the PICU (patient care, systems-based practice, interpersonal and communication skills)

**Summative and formative comments:**

**Milestone assessment:** [Table t4-jetem-7-3-o1]

### Stimulus Inventory

[Table t5-jetem-7-3-o1]

## Stimulus #1

### Patient Information

[Table t6-jetem-7-3-o1]

## Stimulus #2

### Complete Blood Count (CBC)

[Table t7-jetem-7-3-o1]

## Stimulus #3

### Basic Metabolic Panel (BMP)

[Table t8-jetem-7-3-o1]

## Stimulus #4

### Urinalysis

[Table t9-jetem-7-3-o1]

## Stimulus #5

### Venous Blood Gas

[Table t10-jetem-7-3-o1]

## Stimulus #6

### Creatine Kinase (CK)

[Table t11-jetem-7-3-o1]

## Stimulus #7

### Thyroid Panel

[Table t12-jetem-7-3-o1]

## Stimulus #8

### COVID and Influenza Testing

[Table t13-jetem-7-3-o1]

## Stimulus #10

### MRI Cervical, Thoracic, and Lumbar Spine with + without contrast

[Fig f1-jetem-7-3-o1] **Reading: T2-hyperintensity within the anterior spinal cord at the levels of C5 – L4 with selective involvement of the gray matter; Axial T2 weighted image demonstrates prominent anterior horn cell involvement especially on the right in the lower cervical spine and upper thoracic spine. Contrast-enhanced T1 weighted images demonstrate ventral root enhancement in same area.**

## Stimulus #11

### CSF Studies

[Table t14-jetem-7-3-o1]

## DEBRIEFING AND EVALUATION PEARLS

### Acute Flaccid Myelitis (AFM)

1. Pediatric patients presenting with neuromuscular weakness should have an evaluation of ventilation status including a negative inspiratory force (NIF) measurement.
2. Pediatric patients with a NIF of more than −30 cm H2O are at risk for respiratory failure and need administration of ventilatory support.
3. AFM weakness can be asymmetrical and have either ascending or descending progression. Paresthesia symptoms are usually absent. GBS demonstrates symmetric weakness in an ascending pattern and symptoms of paresthesia are frequently present.
4. Lumbar puncture should be performed in the setting of neuromusculoskeletal pathology which will demonstrate pleocytosis usually <100 per μL with lymphocytic predominance which differs from GBS (cell count usually less than 10 per μL with predominantly mononuclear (lymphocytes and monocytes) cells and spinal cord infarction.^1^,^8^
5. MRI abnormality is part of the CDC criteria for the diagnosis of AFM; while MRI can be used to supplement the diagnosis of GBS, it is not part of the criteria necessary to confirm the diagnosis.^10^
