# Supplementary material for: Acute Flaccid Myelitis
Source: J Educ Teach Emerg Med. 2022 Jul 15;7(3):O1–O28. doi: 10.21980/J8MP9G (PMC10332698; doi:10.21980/J8MP9G)
Supplement: Supplementary file 1 [file jetem-7-3-o1-supp1.pptx]

## Slide 1
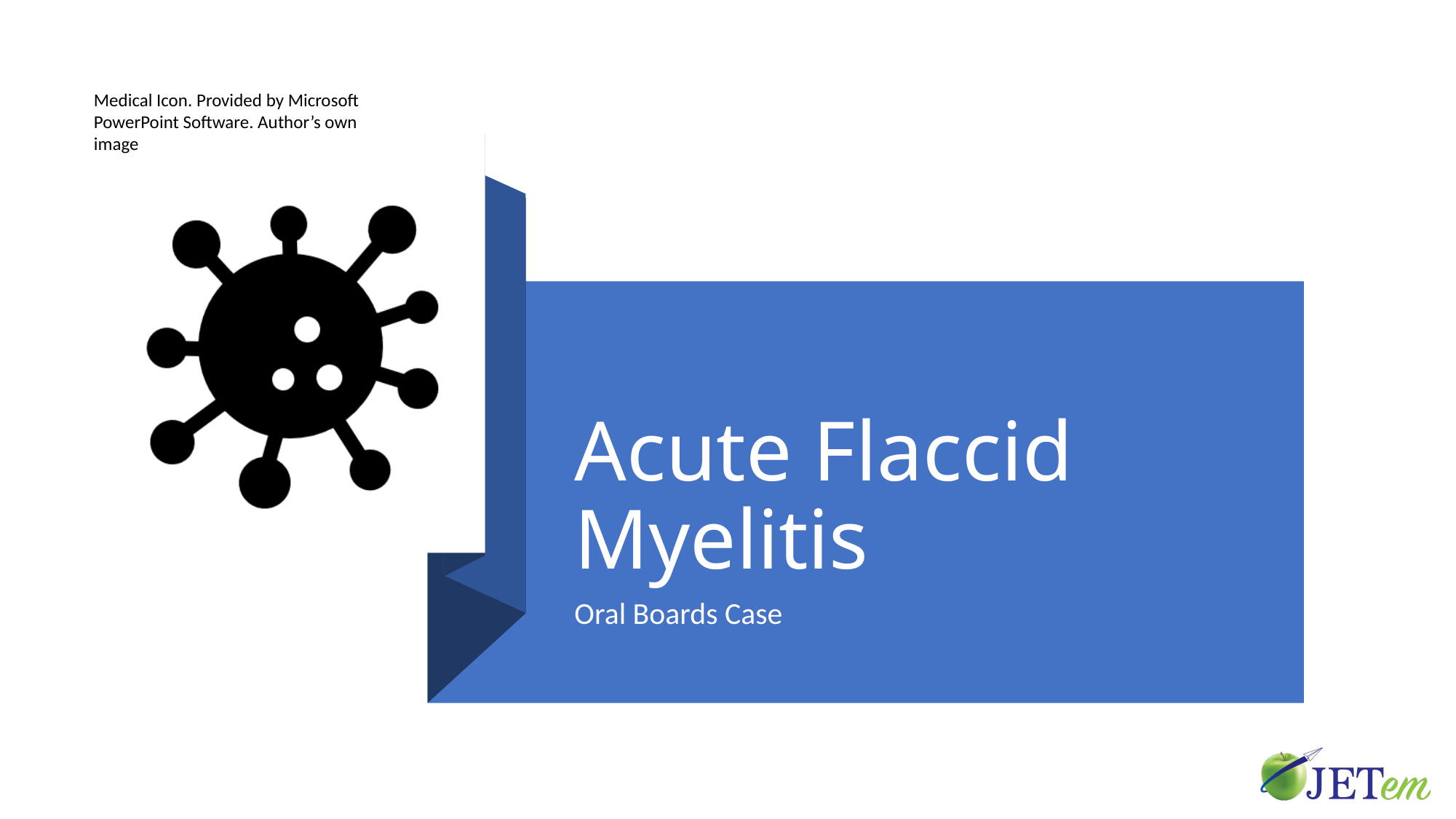

Medical Icon. Provided by Microsoft PowerPoint Software. Author’s own image
# Acute Flaccid Myelitis
Oral Boards Case

## Slide 2
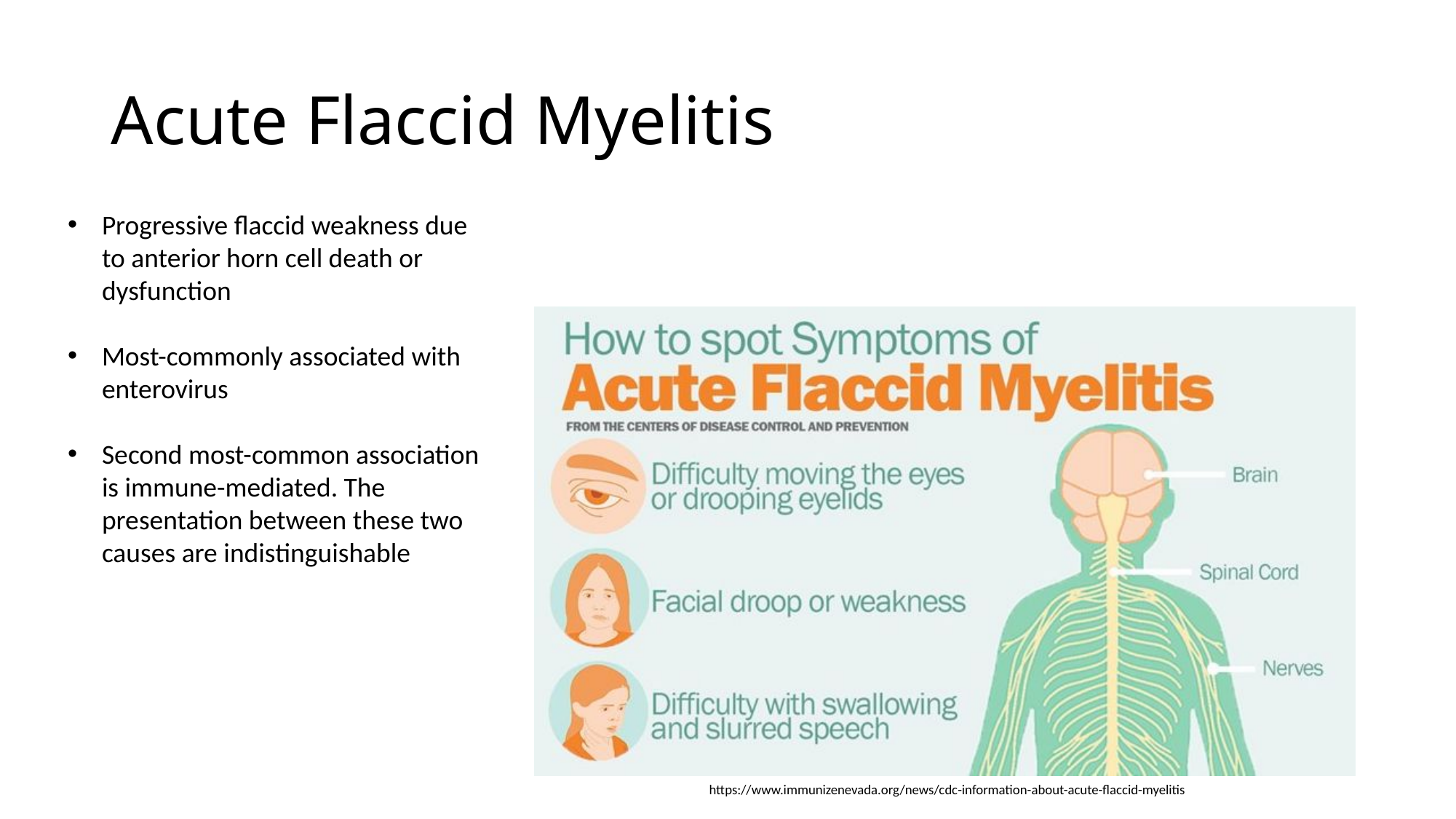

# Acute Flaccid Myelitis
Progressive flaccid weakness due to anterior horn cell death or dysfunction
Most-commonly associated with enterovirus
Second most-common association is immune-mediated. The presentation between these two causes are indistinguishable
https://www.immunizenevada.org/news/cdc-information-about-acute-flaccid-myelitis

## Slide 3
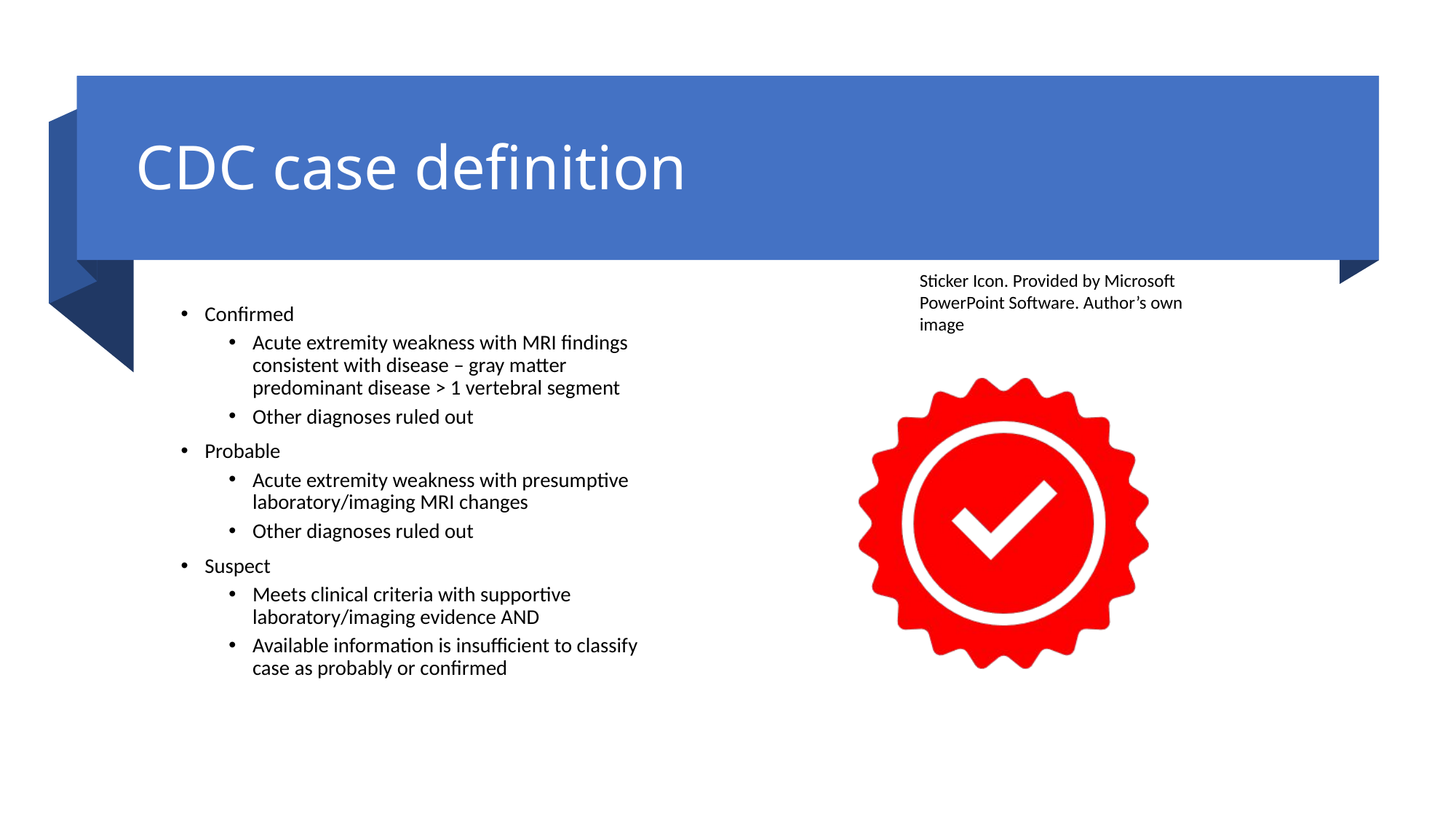

# CDC case definition
Sticker Icon. Provided by Microsoft PowerPoint Software. Author’s own image
Confirmed
Acute extremity weakness with MRI findings consistent with disease – gray matter predominant disease > 1 vertebral segment
Other diagnoses ruled out
Probable
Acute extremity weakness with presumptive laboratory/imaging MRI changes
Other diagnoses ruled out
Suspect
Meets clinical criteria with supportive laboratory/imaging evidence AND
Available information is insufficient to classify case as probably or confirmed

## Slide 4
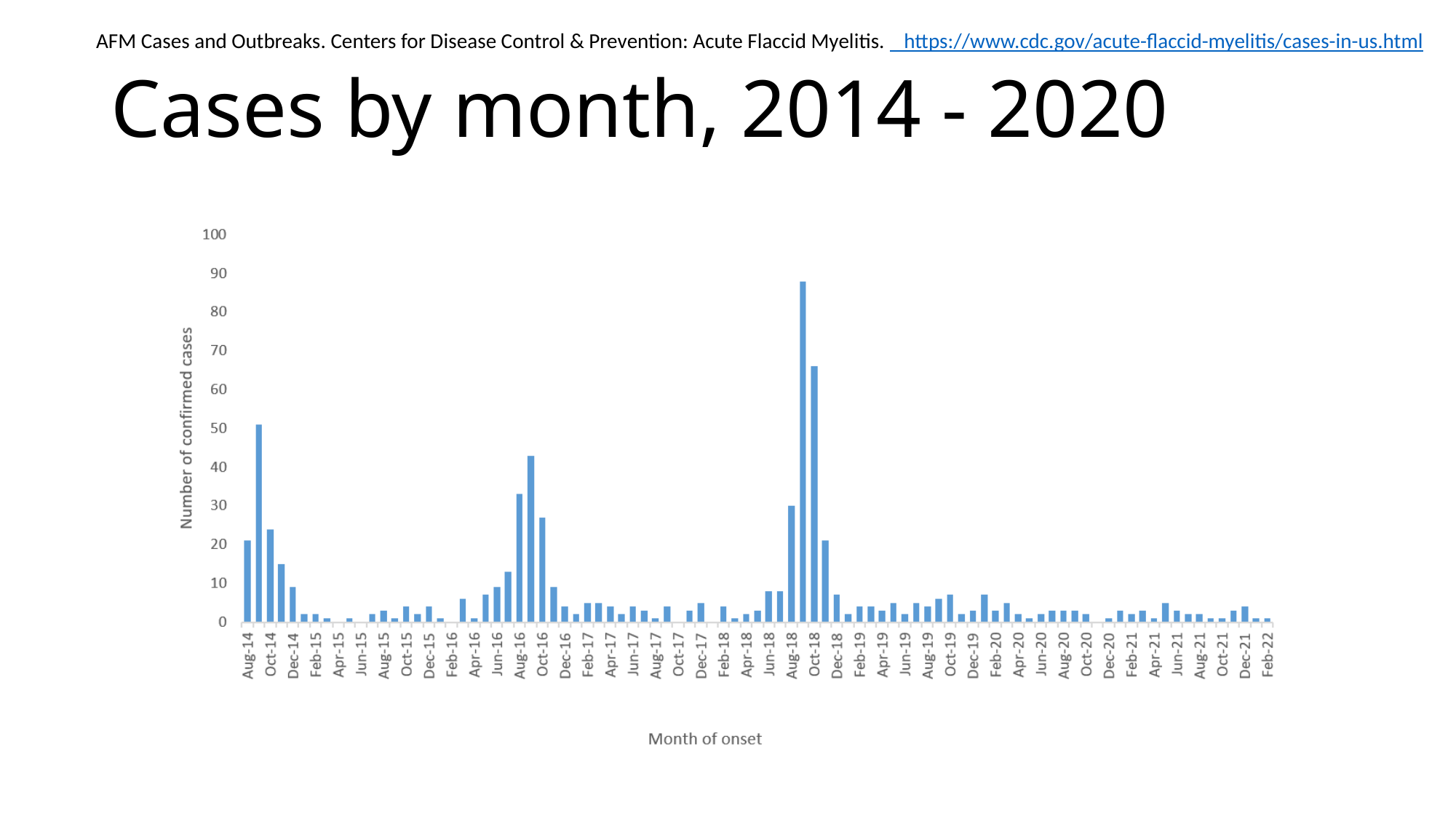

AFM Cases and Outbreaks. Centers for Disease Control & Prevention: Acute Flaccid Myelitis. https://www.cdc.gov/acute-flaccid-myelitis/cases-in-us.html
# Cases by month, 2014 - 2020

## Slide 5
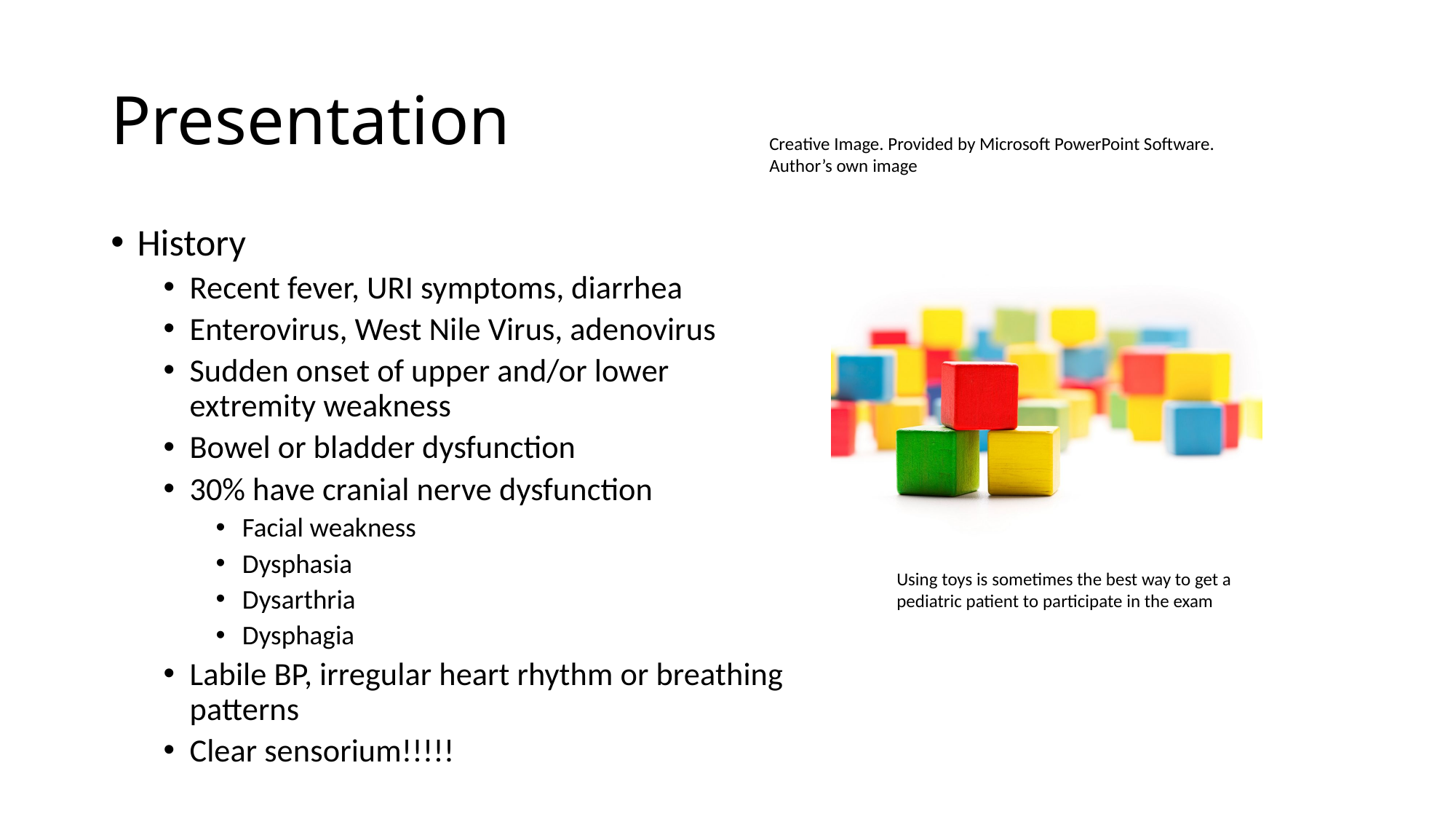

# Presentation
Creative Image. Provided by Microsoft PowerPoint Software. Author’s own image
History
Recent fever, URI symptoms, diarrhea
Enterovirus, West Nile Virus, adenovirus
Sudden onset of upper and/or lower extremity weakness
Bowel or bladder dysfunction
30% have cranial nerve dysfunction
Facial weakness
Dysphasia
Dysarthria
Dysphagia
Labile BP, irregular heart rhythm or breathing patterns
Clear sensorium!!!!!
Using toys is sometimes the best way to get a pediatric patient to participate in the exam

## Slide 6
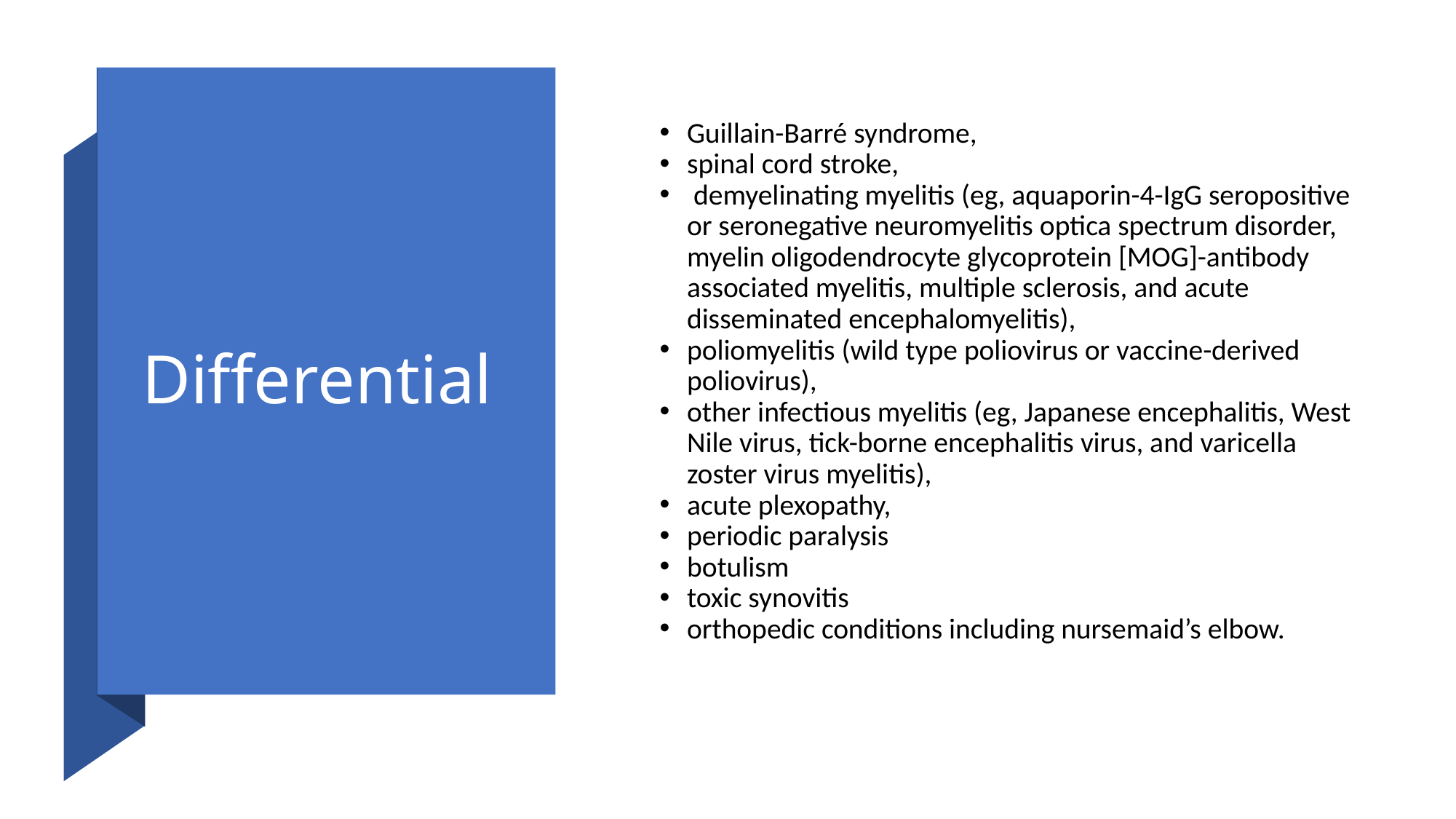

# Differential
Guillain-Barré syndrome,
spinal cord stroke,
 demyelinating myelitis (eg, aquaporin-4-IgG seropositive or seronegative neuromyelitis optica spectrum disorder, myelin oligodendrocyte glycoprotein [MOG]-antibody associated myelitis, multiple sclerosis, and acute disseminated encephalomyelitis),
poliomyelitis (wild type poliovirus or vaccine-derived poliovirus),
other infectious myelitis (eg, Japanese encephalitis, West Nile virus, tick-borne encephalitis virus, and varicella zoster virus myelitis),
acute plexopathy,
periodic paralysis
botulism
toxic synovitis
orthopedic conditions including nursemaid’s elbow.

## Slide 7
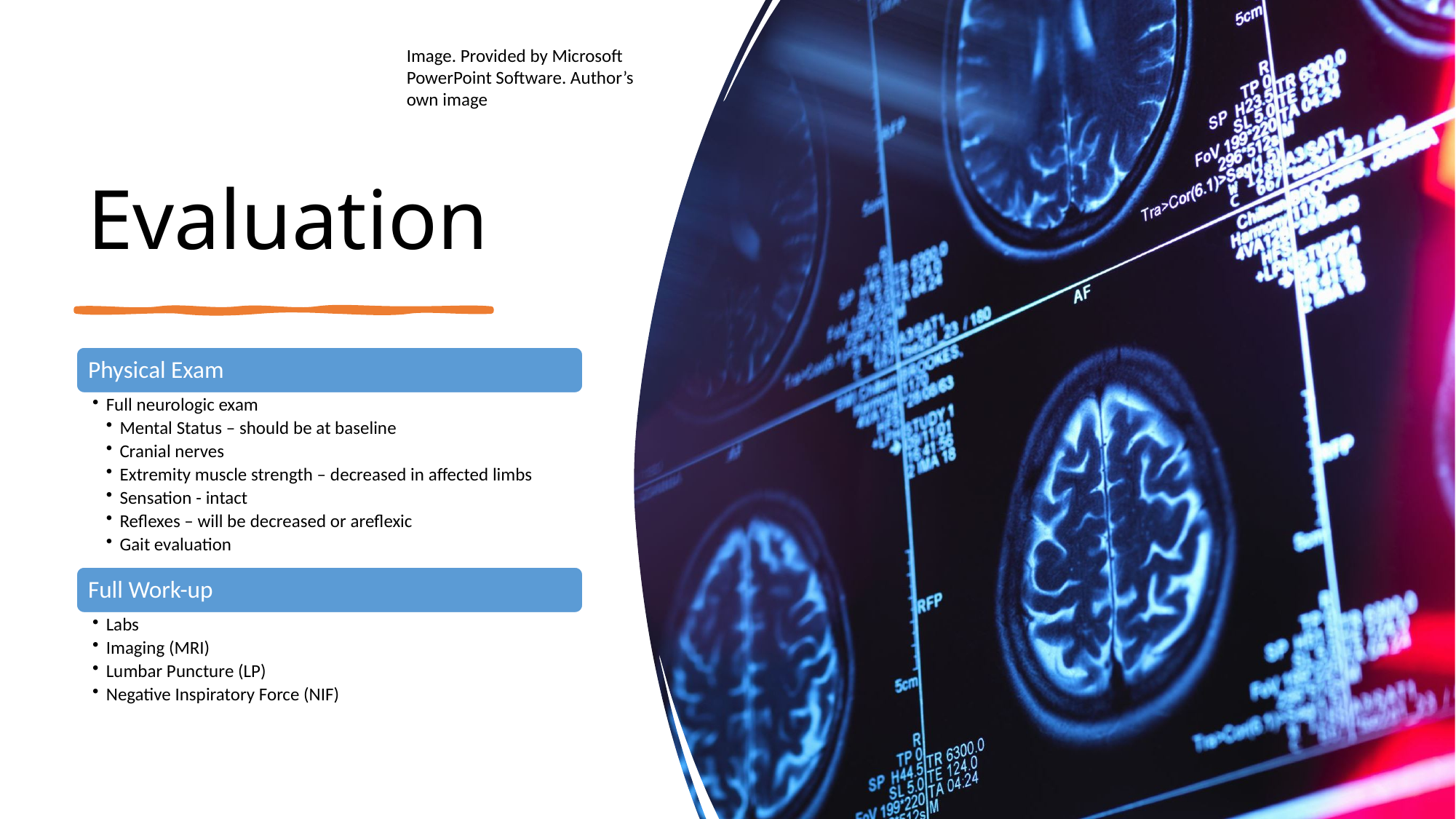

# Evaluation
Image. Provided by Microsoft PowerPoint Software. Author’s own image

## Slide 8
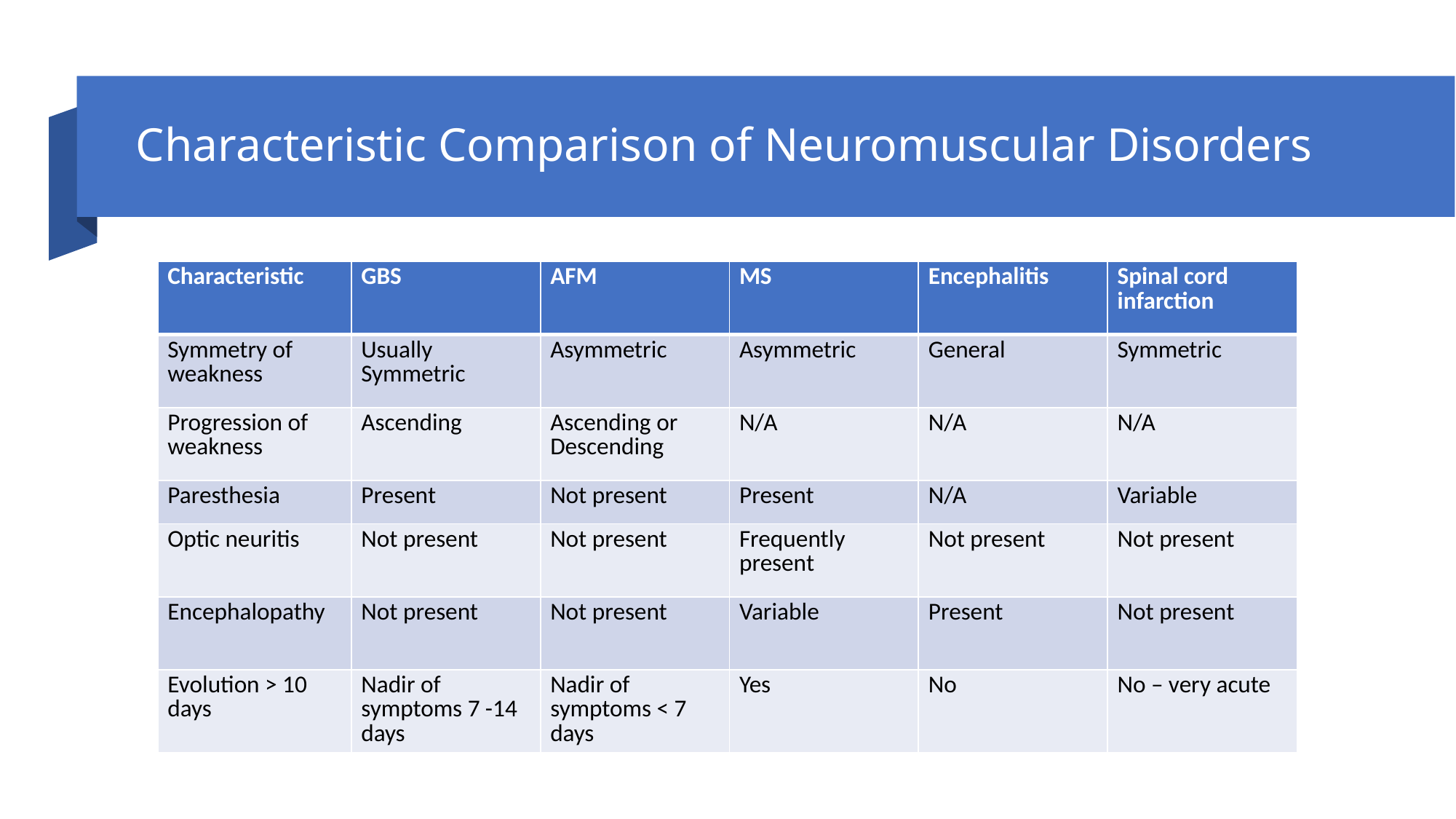

# Characteristic Comparison of Neuromuscular Disorders
| Characteristic | GBS | AFM | MS | Encephalitis | Spinal cord infarction |
| --- | --- | --- | --- | --- | --- |
| Symmetry of weakness | Usually Symmetric | Asymmetric | Asymmetric | General | Symmetric |
| Progression of weakness | Ascending | Ascending or Descending | N/A | N/A | N/A |
| Paresthesia | Present | Not present | Present | N/A | Variable |
| Optic neuritis | Not present | Not present | Frequently present | Not present | Not present |
| Encephalopathy | Not present | Not present | Variable | Present | Not present |
| Evolution > 10 days | Nadir of symptoms 7 -14 days | Nadir of symptoms < 7 days | Yes | No | No – very acute |

## Slide 9
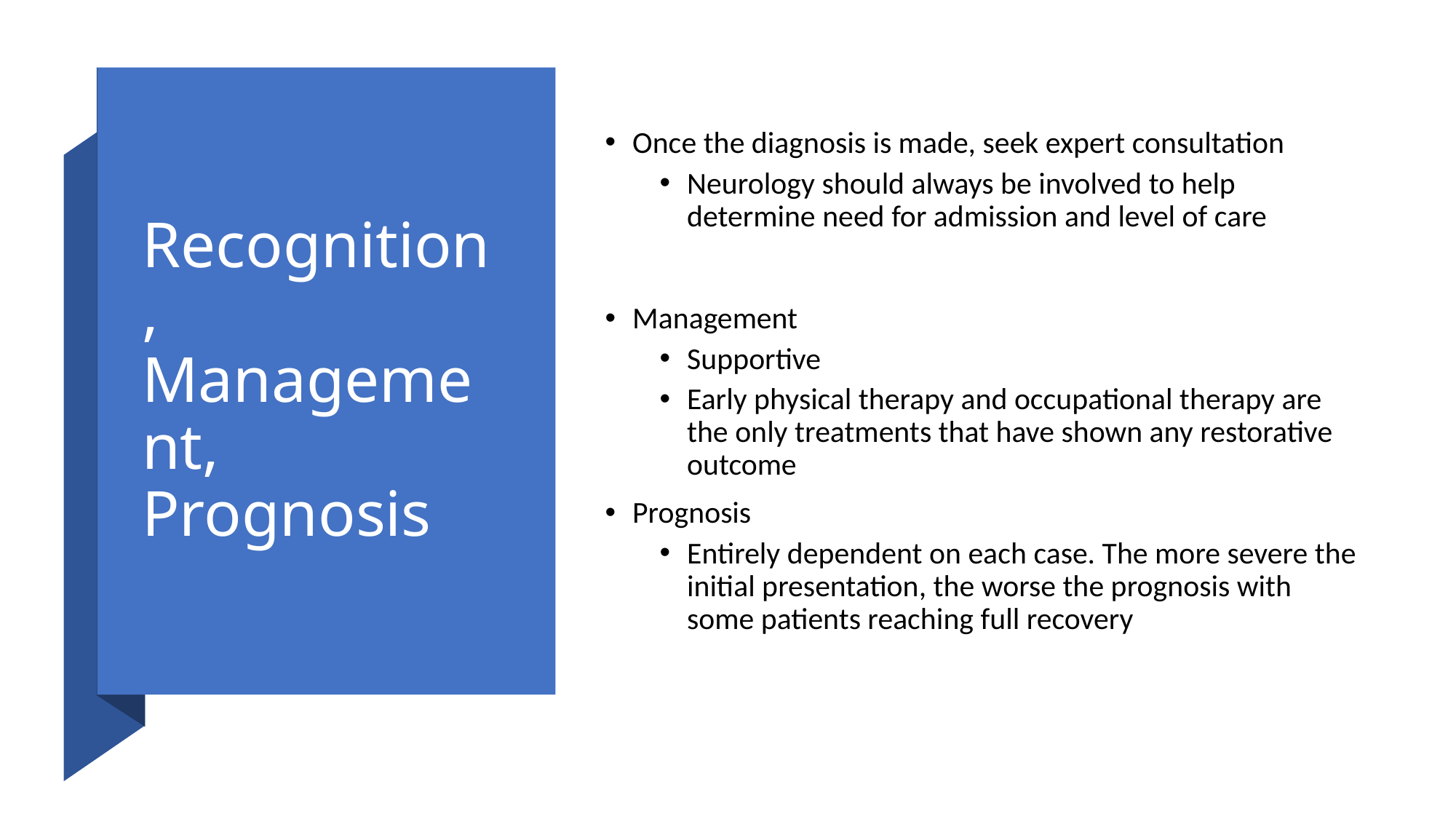

# Recognition, Management, Prognosis
Once the diagnosis is made, seek expert consultation
Neurology should always be involved to help determine need for admission and level of care
Management
Supportive
Early physical therapy and occupational therapy are the only treatments that have shown any restorative outcome
Prognosis
Entirely dependent on each case. The more severe the initial presentation, the worse the prognosis with some patients reaching full recovery
